# Supplementary material for: Identification of photoperiod-induced specific miRNAs in the adrenal glands of Sunite sheep (Ovis aries)
Source: Front Vet Sci. 2022 Jul 22;9:888207. doi: 10.3389/fvets.2022.888207 (PMC9354845; doi:10.3389/fvets.2022.888207)
Supplement: Supplementary file 1 [file Data_Sheet_1.ZIP › Supplementary file/miRDeep2 file.zip!/miRDeep2 file/Novel_Structure/LP42A3/Novel_460.pdf]

Provisional ID	:	NC_019464.2_9853
Score total	:	2.1
Score for star read(s)	:	-1.3
Score for read counts	:	0
Score for mfe	:	1.8
Score for randfold	:	1.6
Score for cons. seed	:	
Total read count	:	55
Mature read count	:	55
Loop read count	:	0
Star read count	:	0

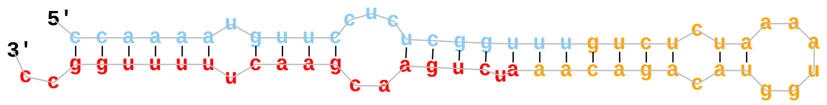

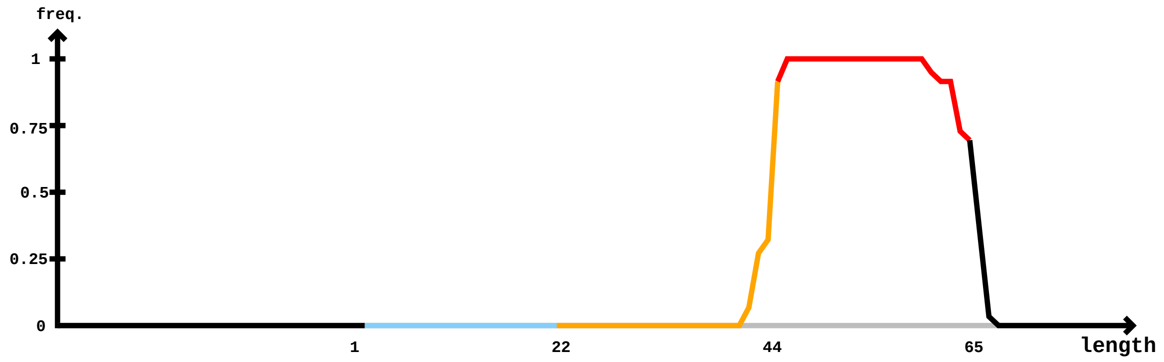


```
                                                  Star                                       Mature
5'- aaacugaccuuuuccauuguauuggguuggccaaaauguuccucucgguuugucucuaaaaugguacagacaaaucugaacgaacuuuuuggccaacccaaacuuccuug   -3'    exp
    ..................((.(((((((((((((((.((((...(((((((((((.((......)).))))))).))))..)))).)))))))))))))))))).......   reads      mm         sample
    ......................................................................Aaaaucugaacgaacuuuu.....................   3          1          seq
    ......................................................................caaaucugaacgaacuuuuu....................   1          0          seq
    .......................................................................aaaucugaacgaacuuuuC....................   1          1          seq
    .......................................................................aaaucugaacAaacuuuuugg..................   11         1          seq
    ........................................................................aaucugaacAaacuuuuuggc.................   1          1          seq
    ........................................................................aaucugaacAaacuuuuuggcc................   2          1          seq
    .........................................................................aucugaacAaacuuuuuggcc................   34         1          seq
    .........................................................................aucugaacAaacuuuuuggccaa..............   1          1          seq
    ..........................................................................ucugaacAaacuuuuuggc.................   1          1          seq
    ..........................................................................ucugaacgaacuuuuuggcU................   1          1          seq
    ..........................................................................ucugaacAaacuuuuuggcca...............   2          1          seq
    ..........................................................................ucugaacgaacuuuuuggccaa..............   1          0          seq
```
